# Determinants of post cesarean section surgical site infection at public hospitals in Dire Dawa administration, Eastern Ethiopia: Case control study

**DOI:** 10.1371/journal.pone.0250174

**Published:** 2021-04-16

**Authors:** Samuel Dessu, Serawit Samuel, Feleke Gebremeskel, Alemu Basazin, Zerihun Tariku, Meles Markos

**Affiliations:** 1 Department of Public Health, College of Medicine and Health Sciences, Wolkite University, Wolkite, Ethiopia; 2 Department of Public Health, College of Medicine and Health Sciences, Wolaita Soddo University, Soddo, Ethiopia; 3 School of Public Health, College of Medicine and Health Sciences, Arba Minch University, Arba Minch, Ethiopia; 4 Department of Nursing, College of Medicine and Health Sciences, Debre Berhan University, Debre Berhan, Ethiopia; 5 Department of Public Health, College of Medicine and Health Sciences, Dire Dawa University, Dire Dawa, Ethiopia; University of Mississippi Medical Center, UNITED STATES

## Abstract

**Introduction:**

Post cesarean section surgical site infection increases both the duration of a patient’s hospital stay and unplanned hospital costs. It can delays recovery, prolongs hospitalization, necessitates readmission, and adds to hospital bills and other morbidities as well as mortalities.

**Method:**

Facility-based case-control study was conducted from 1^st^ March to 20^th^ April, 2019 among all the mother records enrolled from 1^st^ January to 31^st^ December, 2018 at Public hospitals in Dire Dawa administration. The records of the mothers’ who had post-cesarean section surgical site infection (119) was extracted by a census and every three consecutive controls (357) for each case were collected by trained data collectors using a structured data extraction tool. Variables which had p-value <0.25 in bivariate analysis were considered as candidates for multivariable analysis. Statistical significance was declared at P-value ≤0.05 with adjusted odd ratio and 95% confidence interval in the multivariable logistic regression model.

**Result:**

Age 20–34 years (AOR:5.4; 95%CI:2.35,12.7), age >35 years (AOR:8.9; 95%CI:1.8,43.9), ≥4 per vaginal examinations (AOR: 4.2; 95%CI:2.16,8.22), current history of Chorioamnionitis (AOR:5; 95%CI:1.05,23.9), previous history of cesarean section (AOR:6.2; 95%CI: 2.72,14.36), provision of antibiotics prophylaxis (AOR:3.2; 95%CI:1.81,5.62), perioperative HCT level <30% (AOR:6.9; 95%CI:3.45,14.1) and duration of rupture of membrane >12 hours (AOR:5.4; 95%CI:1.84,15.87) were the independent determinants of post-cesarean section surgical site infection.

**Conclusion:**

Increased in age of the mother, higher number of per vaginal examination, having a history of chorioamnionitis, having previous history of cesarean section, not receiving antibiotics prophylaxis, lower perioperative hematocrit level and longer duration of rupture of membrane were statistically significant in multivariable analysis. Therefore; emphasis should be given for mothers who have higher age category, previous cesarean scar and history of choriamnionitis. In addition; provision of antibiotics should be comprehensive for all mothers undergoing cesarean section.

## Introduction

A cesarean section (CS) is a procedure in which surgery was made through a mother’s abdominal wall and underlying tissues to expel the baby [1]. It is the most commonly performed major abdominal operations among women in both developed and developing countries [2]. Despite its benefit, it can results in infection, postpartum hemorrhage, bladder injury and increased risks during future pregnancies [3].

Post-cesarean section surgical site infection is a term used to describe an infection that happens within the first 30 days after surgery of abdominal skin and the underlying tissues [4]. It is the second most commonly occurring infectious complication next to urinary tract infection following cesarean section [5]. It is a known cause of maternal morbidity and mortality [6].

The magnitude of post cesarean section surgical site infection has been varying according to the population being studied, the methods used to monitor and identify cases, and the use of appropriate antibiotic prophylaxis [7, 8]. Globally, post CS SSIs rate differ substantially and are higher in less developed countries, compared to more developed countries where advanced hospital infection control services exist and correct implementation of evidence–based guidelines for SSI prevention are functional [9]. In Sub Saharan countries, SSI is the commonest infection in the patients affecting up to two thirds of patients undergoing surgery [10].

The global prevalence of SSI after cesarean section varied from 3% to15%, depending on the surveillance methods used to identify infections, the patient population, and the use of antibiotic prophylaxis [9]. It increases both the duration of a patient’s hospital stay and unplanned hospital costs [11]. It can delay the recovery, prolongs hospitalization, necessitates readmission, and adds to hospital bills and other morbidities as well as mortalities [12].

Research reports illustrated that; pregnancy induced hypertension, prolonged labor duration, type of surgery, prolonged operation time, multiple vaginal examinations during labor, chorio-amnionitis, presence of meconium, large intraoperative blood loss and Perioperative blood transfusion, younger age and premature rupture of the membranes were significantly associated with post caesarean section surgical site infection [13–17].

In Ethiopia, the magnitude of post cesarean SSI was ranged from 8.81% to15% [18–20] and certain factors associated with post-cesarean surgical site infection were identified through cross-sectional studies such as age, hypertensive disorders of pregnancy, duration of labor, prophylaxis provision and wound classification. Therefore, this study aims to assess the determinants of post-cesarean section surgical site infection among mothers delivered at Governmental hospitals in Dire Dawa administration, Eastern Ethiopia. In addition; this study will be an input for policy implementation together with the studies conducted in Ethiopia before.

## Methods

### Study design, area and period

A case-control study was conducted at Dire Dawa administration Public Hospital from 1^st^ March to 20^th^ April 2019 among the records enrolled from 1^st^ January to 31^st^ December, 2018. Dire Dawa is located at the Eastern part of Ethiopia which is 515km far from Addis Ababa, the capital city of Ethiopia. The hypothesis questioned for this study was “mothers diagnosed with post-cesarean section surgical site infection are likely to be exposed to a variety of different exposures than those mothers not having post-cesarean section surgical site infection”

### Populations

**Fig 1:**
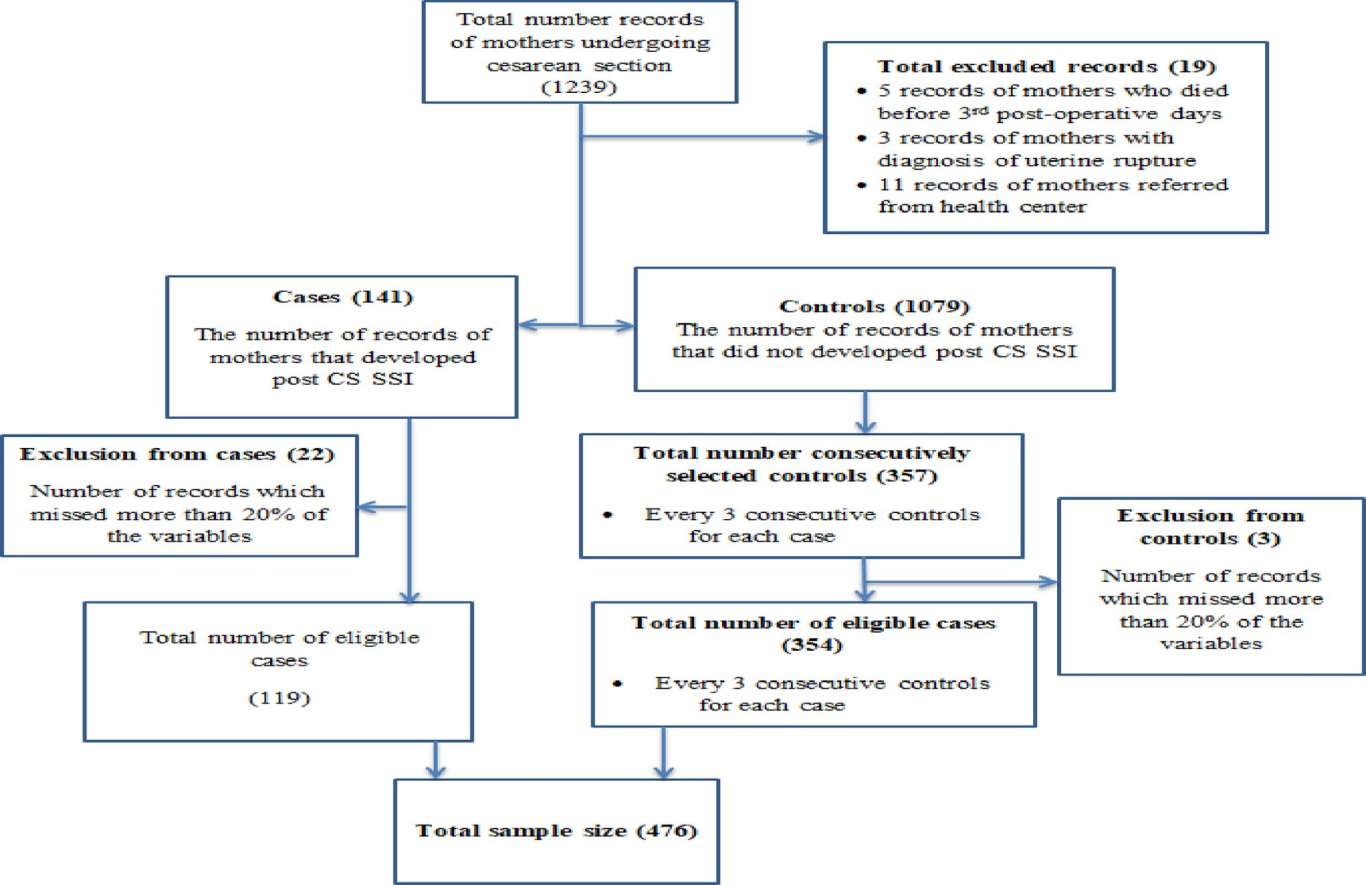
Schematic presentation of sampling method for the study on determinants of post cesarean section surgical site infection at public hospitals at Dire Dawa administration, Eastern Ethiopia.

Women who died before 3^rd^ post-operative days, women with a diagnosis of uterine rupture and women referred from other health facilities for further investigation or diagnosis of surgical site infection were excluded from this study. Within this time frame, there were 119 cases that fulfill the eligibility criteria and for each case, 3 consecutive controls were taken. Therefore, this study was conducted with a total of 476 (119 cases and 357 controls) study subjects (Fig 1).

### Study variables

The outcome variable was post-cesarean section surgical site infection and the independent variables were classified as maternal factors (age, duration of stay in hospital, wound class, level pre-operative hematocrit level), medical Co morbidities (hypertension, Diabetes Mellitus), obstetrical Factors (nulliparity, premature rupture of membrane, prolonged rupture membrane, duration of labor, chorioamnionitis, twin gestation, previous Cesarean scar) and procedure related factors (digital examination, perioperative blood transfusion, antibiotic as a prophylaxis, emergency cesarean section, type of incision, incision length, duration of procedure).

### Operational definition

Post-cesarean section surgical site infection: An infection that happens within the first 30 days after surgery of abdominal skin and the underlying tissues (4).

Case: A record of a mother who delivered by cesarean section and who had developed SSI prior to hospital discharge.

Control: A record of a mother who delivered by cesarean section and who had not developed and SSI prior to hospital discharge.

### Data collection tool, procedure and quality assurance

The sources of data were individual patient records including registers, monitoring cards, and patient admission books. Records that missed more than 20 percent of the variables were excluded before data collection. Data was collected using a structured checklist or questionnaire which was prepared globally by WHO. Data were collected by trained health professionals (researchers).

Pretest was performed by 5% of the population to check the consistency of the data collection tool and to check the competency of the trained data collectors. A sensitivity analysis was conducted and which was 78%. In addition, content validity was cross-checked by another reproductive health expert.

### Data processing and analysis

Data were cleaned, coded and entered into Epi Data version 3.0.1 and exported to SPSS version 25 for Windows, and then exploratory data analysis was carried out to check the levels of missing values, presence of influential outliers and multicolinearity. Crude odds ratios and AOR with 95% confidence interval were computed to assess the presence of a degree of association between the variables.

Both bivariate and multivariable logistic regression models were fitted to assess the association between outcome and explanatory variables. Independent variables that have p-value <0.25 in bivariate logistic regression were entered into the multivariable logistic regression model. The extent of strength was presented using odds ratios and its 95% confidence intervals. P-value ≤ 0.05 was used as a cutoff point to determine statistical significance in multivariable logistic regressions model.

### Ethical consideration

Ethical clearance was obtained from the Dire Dawa University, college of medicine and health sciences research and ethical review board to conduct the study as verified in ethics approval letter reference number DDU/RTI/036/19. Since, the study was conducted through a record review, the study participants were not available at the moment and the responsibility was kept upon the respective hospital directors. Therefore; further permission was obtained from Dire Dawa health bureau and medical director of the selected hospitals.

## Results

### Maternal and procedure related factors

**Fig 2:**
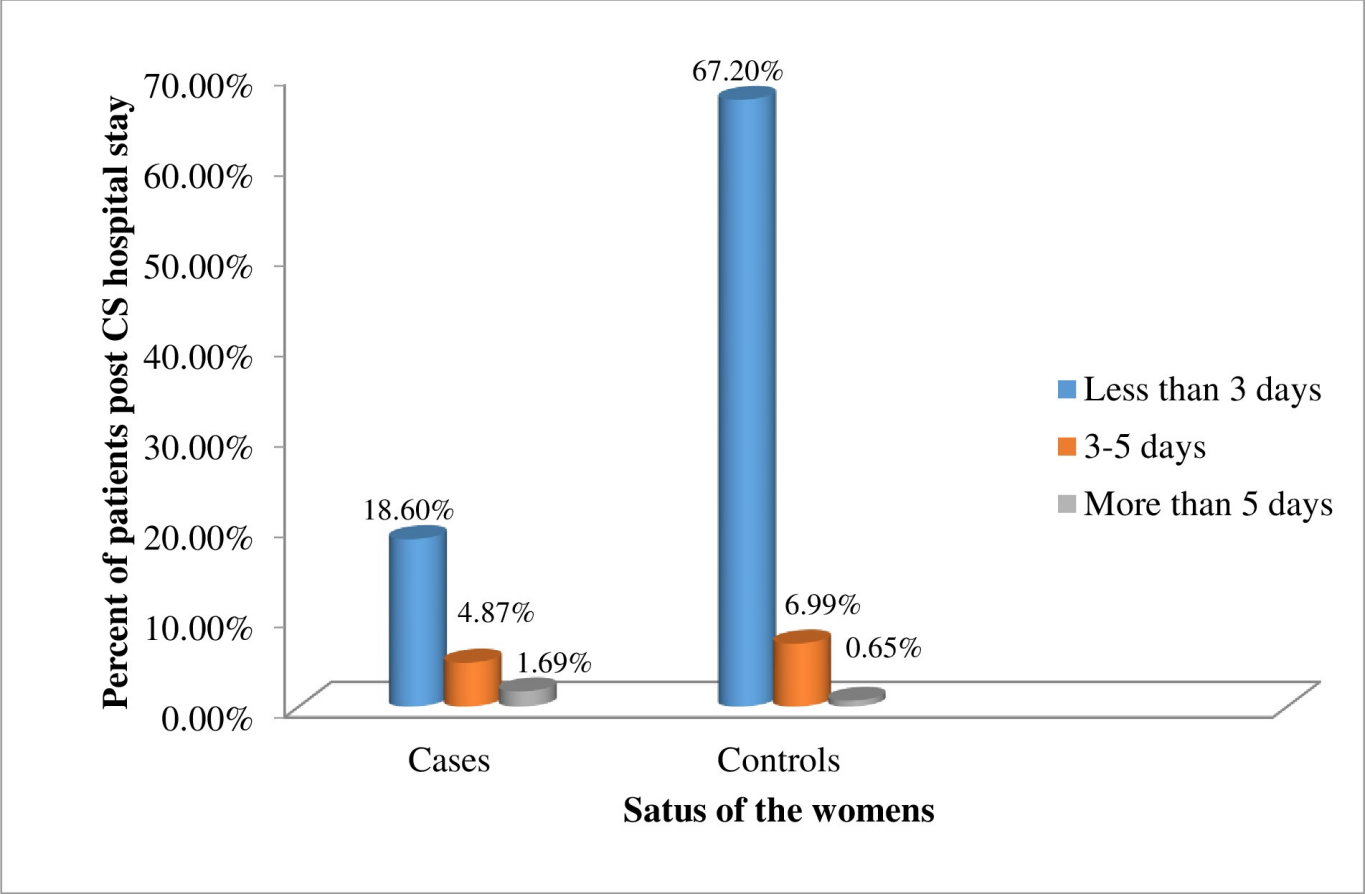
Proportion of women with post cesarean section hospital stay at Dire Dawa public hospitals, Eastern Ethiopia.

The minimum and maximum duration of post-cesarean section hospital stay for the cases was one day and eight days respectively with the mean of 3.76±1.97 days while it was one day and six days respectively for the control groups with a mean of 2.67±1.55 days. Majority of the respondents among the cases (18.6%) and 317(67.2%) among the controls were admitted for less than 3 days postoperatively while 56(11.9%) and 11(2.3%) of the mothers were admitted for three to five days and more than five days respectively (Fig 2).

The minimum age of the cases was 18 years old and the maximum age was 41 years old with the mean age of 30.5±6.36 years old whereas the minimum and maximum age of the controls was 18 and 35 years old respectively with the mean age of 25.14±5.5 years old. Less than one-fifths of the respondents (18%) among the cases and 65.9% among the controls were found in the age range of 20–34 years old while the small proportion among the cases (0.85%) and controls (3.81%) were found at the age of 19 years old and below.

Regarding the history of peri procedure blood transfusion, 5(1.1%) of the cases and 24(5.1%) of the controls received blood transfusion perioperatively. Among those for whom blood transfused, 4(14.3%) of the cases and 13(46.2%) of the controls received one unit of blood and the remaining 1(3.7%) of the cases and 10(35.8%) of the controls received two units of blood. Nearly one-tenths (11.4%) of the cases and 226(47.9) of the controls receive antibiotics as a prophylaxis.

The minimum and maximum amount of the pre-operative hematocrit level among the cases was 21% and 46% with the mean of 35.3±5.2% while it was 23% and 48% among the controls respectively with the mean of 37.9±4.3%. Among them 32(6.8%) of the cases and 28(5.9%) of the controls had a pre-operative hematocrit below 30% (**Table 1**).

**Table 1:** Maternal and procedure related factors of mothers with post cesarean section surgical infection at Dire Dawa administration public hospitals, Eastern Ethiopia.

Variables	Category	Status of respondent		P-value
		Cases(n = 119)	Control(n = 354)	
		n(%)	n(%)	
Age(Year)	<19	4(3.4%)	18(5.1%)	0.0001
	20–34	85(71.4%)	311(88.1%)	
	>35	30(25.2%)	24(6.8%)	
Received Per vaginal examination practice		116(97.5%)	351(99.4%)	0.072
Number of Per vaginal examinations	1–3	84(70.6%)	314(88.9%)	0.0001
	Four and above	35(29.4%)	39(11.1%)	
Type of cesarean section	Emergency	24(20.2%)	69(19.5%)	0.883
	Elective	95(79.8%)	284(80.5%)	
Received Blood transfusion		5(4.2%)	24(6.8%)	0.308
Received Antibiotics prophylaxis		54(45.4%)	226(64.1%)	0.0001
Pre-operative hematocrit level (%)	<30	32(26.9%)	28(7.9%)	0.0001
	>30	87(73.1%)	325(92.1%)	

### Obstetrics and related factors of post cesarean section surgical site infection

The minimum and maximum gestational age of the cases was 32 weeks and 41 weeks respectively with the mean of 37.1±2.66 weeks while the minimum and maximum weeks of gestation among the controls were 32 weeks and 42 weeks respectively with the mean of 37.7±2.38 weeks. Nearly one fifth (19.1%) of the cases and 282(59.6%) of the controls were delivered at 36 weeks and above gestational week. Nearly for on fourth (24.6%) of the cases and three fourth (74.4%) of the controls per vaginal examination were performed. Among those for whom per vaginal examination was performed, 84(17.8%) of the cases and 314(66.5%) of the controls, a total of three and below per vaginal examinations were performed.

Regarding the current history of chorioamnionitis, nine (1.9%) of the cases, and five (1.1%) of the controls experienced chorioamnionitis. Nearly one fourths (22.5%) of the cases and three fourths (74.2%) of the controls had a single tone of pregnancy and 12(2.5%) and 2(0.4%) of the cases and controls had twin pregnancies respectively. Regarding the previous history of cesarean-section, 33(7%) of the cases, and 20(4.2%) of the controls experienced a previous history of cesarean section. In considering the type of the current cesarean section, for one fifths (20.1%) of the cases and 284(60.2%) of the controls elective cesarean section was performed.

The minimum and maximum duration of labor for the cases was 1 hour and 48 hours respectively, with the mean of 9.69±8.94 hours while the minimum duration of labor for the controls was 1 hour and the maximum duration of labor was 24 hours with a mean of 8.56⩲5.49 hours. The majority of the respondents among the cases (21.1) and 298(63.1%) of the controls had a labor duration of 12 hours and below. In addition; 4(0.8%) of the cases and 1(0.2%) of the controls had a labor duration of more than 24 hours.

The minimum and maximum duration of the ruptured membrane among the cases was 1 hour and 48 hours respectively, with the mean of 16.6±20.8 hours while the minimum and the maximum duration of the ruptured membrane among the control groups was one hour and 9 hours respectively with the mean of 1.8±1.6 hours. Nearly one fifths (20.3%) of the cases and 338(71.6%) of the controls had duration of rupture of membrane 12 hours and below. Regarding the total number of pregnancies, 21(4.4%) of the cases and 39(8.3%) of the controls had more than five instances of pregnancies (Table 2).

**Table 2:** Obstetric and related characteristics of the mothers with post-operative surgical site infection at public hospitals of Dire Dawa administration, 2019.

Variables	Category	Status of the respondent		P-value
		Cases(n = 119)	Controls(n = 354)	
		n (%)	n (%)	
Gestational age(weeks)	<34	7(5.9%)	5(1.4%)	0.027
	34–36	22(18.5%)	66(18.7%)	
	>36	90(75.6%)	282(79.9%)	
Having history of Chorioamnionitis?		9(7.6%)	5(1.4%)	0.001
Number of fetuses	Single	106(89.1%)	350(99.2%)	0.304
	Twins	12(10.1%)	2(0.6%)	
	Triples	1(0.8%)	1(0.3%)	
Having Previous history of cesarean section		33(27.7%)	20(5.7%)	0.0001
Duration of labor(hrs)	<12	99(83.2%)	298(84.4%)	0.170
	12–24	16(13.4%)	54(15.3%)	
	>24	4(3.4%)	1(0.3%)	
Duration of ROM	<12	96(80.7%)	338(95.8%)	0.0001
	>12	23(19.3%)	15(4.2%)	
Gravidity	1–4	98(82.4%)	314(88.9%)	0.355
	>5	21(17.6%)	39(11.1%)	

### Determinants of post cesarean section surgical site infection

The multivariable analysis revealed that the age of the women, the number of per vaginal examinations performed, history of Choriamnionitis, history of previous cesarean section, antibiotic prophylaxis provision, preoperative hematorit level and duration of the rupture of the membrane were statistically significant.

Women having age between 20–34 years old were 5 times more likely to have post cesarean surgical site infection compared with those of women who had age 19 years and below(AOR: 5.4; 95%CI: 2.35, 12.7). The odds of the likelihood of occurrence of post-cesarean section surgical site infection among mothers having age 35 years old and above was 9 times more likely compared with women who had age 19 years and below(AOR: 8.9; 95%CI: 1.8,43.9).

The likelihood of the occurrence of post-cesarean section surgical site infection among women who had four and above per vaginal examination was 4 times more likely as compared with the counterparts 1 to 3 per vaginal examinations(AOR: 4.2; 95%CI: 2.16, 8.22). Women who had previous history of Choriamnionitis were 5 times more likely to have post-cesarean section surgical site infection as compared with the counterparts who had no previous history of Chorioamnionitis(AOR: 5; 95%CI: 1.05, 23.9). Women who had previous history of cesarean section had 6 times more likely to have post-cesarean section surgical site infection as compared with those of who had no previous history(AOR: 6.2; 95%CI: 2.72, 14.36).

The odd of the likelihood of the occurrences of post-cesarean section surgical site infection among mothers who had not taken antibiotics as a prophylaxis were 3 times more likely as compared with those who had taken antibiotics as a prophylaxis(AOR: 3.2; 95%CI: 1.81, 5.62). Women who had preoperative hematocrit level less than 30% were 7 times more likely to have post-cesarean section surgical site infection as compared with the counterparts who had more than 30%(AOR: 6.9; 95%CI: 3.45, 14.1). The odd of the likelihood of the occurrence of post-cesarean surgical site infection among women with the duration of the rupture of the membrane more than 12 hours is 5 times more likely as compared with those of 12 hours and below(AOR: 5.4; 95%CI: 1.84, 15.87) (Table 3).

**Table 3:** Factors associated with post cesarean section surgical site infection at Dire Dawa administration public hospitals, 2019.

Variables	Category	Status		COR(95%CI)	AOR(95%CI)
		Cases (119)	Control (354)		
Age(Year)	<19	4	18	1	1
	20–34	85	311	1.22(0.27,2.46)	5.4(2.35,12.7)**
	>35	30	24	5.6(0.05,0.59)*	8.9(1.8,43.9)**
Number of Per vaginal examinations	1–3	84	314	1	1
	Four and above	35	39	3.35(0.18,0.49)*	4.2(2.16,8.22)**
History of Choriamnionitis	Yes	9	5	5.69(0.06,0.53)*	5(1.05,23.9)**
	No	110	348	1	1
History of previous Cesarean section	Yes	33	20	6.38(0.09,0.29)*	6.2(2.72,14.36)**
	No	86	333	1	1
Provision of antibiotics prophylaxis	Yes	54	226	1	1
	No	65	127	2.14(0.31,0.71)*	3.2(1.81,5.62)**
Pre-operative hematocrit (%)	<30	32	28	4.26(0.13,0.41)*	6.9(3.45,14.1)**
	>30	87	325	1	1
Duration of rupture of membrane(hrs)	<12	96	338	1	1
	>12	23	15	5.39(0.09,0.37)*	5.4(1.84,15.87)**

* indicates variables which had p-value <0.25, ** indicates variables which had p-value <0.05.

## Discussion

This study was conducted to assess the determinants of post cesarean section surgical site infection at Dire Dawa administration. Age of the mothers becomes one of the determinants of post cesarean section surgical site infection. Consistent with the study conducted at Bugando medical center, Tanzania, Women who had age between 20–34 years old were 5 times more likely to have post-cesarean surgical site infection as compared with women who had age 19 years and below (AOR: 5.4; 95% CI: 2.35, 12.7) [21]. This study finding was inconsistent with the study conducted at Assela referral hospital, Democratic Republic of Congo, and Sierra Leone [15, 22]. In addition, the odds of the likelihood of occurrence of post-cesarean section surgical site infection among mothers having age 35 years old and above were 9 times higher compared with women who had age 19 years and below (AOR: 8.9; 95%CI: 1.8,43.9) [23]. This might be probably due to impaired basic and instrumental activities of daily living, cognitive impairment, frailty, use of multiple medications and nutritional status [24].

High numbers of per vaginal examinations leads to the development of post cesarean section surgical site infection. In line with the study conducted at Bugando medical center, which shows repeated per vaginal examinations was a risk for post cesarean section surgical site infection; this study show that, women who had four and above per vaginal examination were 4 times more likely to develop post cesarean section surgical site infection as compared with the counterparts who have less than four per vaginal examinations (AOR: 4.2; 95%CI: 2.16, 8.22) [21]. This may be due to ascending infection leading to secondary spread to the surgical site.

Having a history of Choriamnionitis through the current birth becomes a determinant of post cesarean surgical site infection. Similar with the study conducted at Ayder comprehensive specialized hospital, Felegehiwot Referral Hospital, Bahir Dar and Nova Scotia, this study finding indicate that, Women who had a history of Choriamnionitis through the current birth were 5 times more likely to develop post cesarean section surgical site infection as compared with the counterparts who had no Choriamnionitis(AOR: 5; 95%CI: 1.05, 23.9) [10, 23, 25]. Similarly, studies conducted at Pennsylvania [26], China [27], Thailand [28] and Burkinafaso [29] indicate consistent result. This might be due to the prolonged labor and rupture of membranes contribute to amniotic fluid colonization from the normal flora of the lower genital tract and lead to surgical wound and peritoneal cavity contamination.

In addition; this might be due to the fact that an infected amniotic fluid may transfer pathogens into cesarean section incisions site and the organisms responsible for chorioaminitis might use metritis as focus of infection and disseminate through systemic circulation to easily establish wound infection [30, 31]. In addition; this might be explained by the fact that intact membrane serves as a barrier to ascending infections from the lower genital tract to the uterine cavity. An additional explanation could be due to iatrogenic contamination of the peritoneum during surgery.

Having previous cesarean scar has an effect in the development of post cesarean section surgical site infection [32]. This might be due to presence of previous scar had intraoperative adhesion, weaker scar and poor healing.

Provision of antibiotics as a prophylaxis has a protective effect against post cesarean section surgical site infection or which is an established protective factor for surgical site infection [33]. Consistent with the study conducted at Brazilian women hospital and Jordanian teaching hospital; this study revealed that, mothers who had not taken antibiotics as a prophylaxis were 3 times more likely to develop post cesarean surgical site infection as compared with those who had provided antibiotics as a prophylaxis(AOR: 3.2; 95%CI: 1.81, 5.62) [34, 35]. This might be due to the reason that perioperative antibiotic prophylaxis (PAP) can reduce the incidence of SSI by providing an adequate level of the antimicrobial agent in the tissues before surgery.

In addition; this might be due to that antibiotics have the potential to reduce the number of bacteria surrounding the incision site. The American Society of Hospital Pharmacists(ASHP) guidelines and several authors concluded that utilizing a single prophylactic dose (regardless of the type of antibiotic) was at least as effective, if not superior, to administering multiple doses in reducing infectious caesarean complications [36, 37]. In addition a recommendation given by Kelley Conroy and Errol Norwitz on a 10 evidence base recommendations to prevent surgical site infection after cesarean delivery stated as ’’antibiotic prophylaxis significantly reduced infectious morbidity when it was given 60 minutes before the skin incision, with no significant effect on neonatal outcome" [38].

Inconsistent with the study conducted at Iraq, this study revealed that, women who had preoperative hematocrit level less than 30% were 7 times more likely to have post cesarean section surgical site infection as compared with the counterparts who had more than 30%(AOR: 6.9; 95%CI: 3.45, 14.1) [39]. This study finding is consistent with the study conducted at Mizan Tepi University teaching hospital [40]. This might be due to the hypo perfusion of the wound secondary to anemia and reduced post-operative ambulation. In addition; this might be due to, intra operative blood loss and the nutritional derangement of the mothers or a secondary to blood loss because low hematocrit level indicates the nutritional derangement [41, 42].

Some other studies indicate that emergency cesarean section has been linked to surgical site infection through more frequent vaginal examinations with greater opportunity for membranes to rupture before delivery, highly urgent operation, less concerns about sterility, and absence of prophylactic antibiotics on time. But which is not statistically significant in this study. This might be due to the effects of uncontrolled confounders. Therefore; meta-analysis need to be considered.

The limitation of this study was, since the study was conducted through record review, some variables related with laboratory tests, socio economic, socio-demographic and service related factors were missed. In other sense, the health care quality was not assessed, which may provide an input for mothers with post cesarean section SSI. Even if we have applied appropriate measures to minimize misclassification, being secondary data may cause misclassification of study subjects. In addition, this study did not ascertain the status of control groups who did not follow the respective hospitals for follow up. But post discharge surveillance is essential to address those groups.

## Conclusion

The multivariable analysis indicate that age of the mother, number of per vaginal examination, current history of Chorioamnionitis, previous history of cesarean section, provision of antibiotics prophylaxis, perioperative hematocrit level and duration of ROM were the independent determinants of post cesarean section surgical site infection. Proper assessment of risk factors that predispose to surgical site infection is critical for the development of strategies for reducing the occurrence of SSI like minimizing the number of vaginal examination and minimizing the time gap between rupture of membrane and delivery.

Despite one thirds of the world population lacks access to essential medicines [43]; governmental and non-governmental stakeholders should apply certain efforts to access prophylactic antibiotics across each health facilities to address for all the mothers undergoing cesarean section. In addition; health professionals should be comprehensive in providing prophylactic antibiotics for mothers undergoing cesarean section. Mothers who have older ages should be trained to minimize pregnancy. Health professionals should strengthen their continuous follow up and SSI risk minimization should be emphasized if they are pregnant such as reducing the number of digital examinations, provision of antibiotics prophylaxis before surgery and shortening the duration of labor if they have developed Choriamnionitis.

## Supporting information

S1 File (DOCX)

S2 File (SAV)

## Abbreviations

ANC: Antenatal Care
BEMoNC: Basic Emergency Maternal and Neonatal Care
CS: Cesarean section
GC: Gregorian calendar
MRN: Medical Record Number
NGOs: Nongovernmental Organizations
OR: Odd Ratio
PROM: Premature rupture of membrane
SDG: Sustainable Development Goals
SSI: Surgical site infection
UNICEF: United Nations International and Children’s Emergency Fund
WHO: World Health Organization
