# Supplementary material for: Determinants of post cesarean section surgical site infection at public hospitals in Dire Dawa administration, Eastern Ethiopia: Case control study
Source: PLoS One. 2021 Apr 16;16(4):e0250174. doi: 10.1371/journal.pone.0250174 (PMC8051775; doi:10.1371/journal.pone.0250174)
Supplement: S1 File — (DOCX) [file pone.0250174.s001.docx]

## Data extraction tool

**Title:** Determinants of post CS surgical site infection at governmental Hospitals in Dire Dawa Administration, Eastern Ethiopia, 2019G.C

- Guide to data collectors: Read the variables and fill the data extraction form carefully by making appropriate circle or writing the response on the space provided accordingly.

| Code | **Variables** | **Categories** | **Skip** |
| --- | --- | --- | --- |
| 101 | Which category does the mother belong? | 1. Case 2. Control |  |
| 102 | Age of the women | ________year |  |
| 103 | Does the mother have comorbid hypertension? | 1. Yes 2. No |  |
| 104 | Which type of hypertension is it? | 1. Pregnancy induced hypertension 2. Chronic hypertension |  |
| 105 | Does the mother have comorbid DM? | 1. Yes 2. No |  |
| 106 | How long does the mother admitted? | ------------- days |  |
| 107 | Does vaginal examination has been done | 1. Yes 2. No |  |
| 108 | If yes how many times ? | __________ |  |
| 109 | How many times have you been pregnant? | _________ |  |
| 110 | Duration of the rupture of membrane before the onset of labour? | ___________hrs |  |
| 111 | What was the duration of labor? | ___________(hr) |  |
| 112 | Does the mother have history of choriamnionitis? | 1. Yes 2. No |  |
| 113 | Number of fetuses? | 1. Single 2. Twin 3. Triple |  |
| 114 | Gestational age until rupture of membrane? | ________wks |  |
| 115 | Does the mother have previous history of cesarean section? | 1. Yes 2. No |  |
| 116 | What type of CS is it? | 1. Emergency 2. Elective |  |
| 117 | What type of incision was made? | 1. Vertical 2. Transverse 3. Any other |  |
| 118 | How many hours does the procedure take? | _______min |  |
| 119 | Does the mother take perioperative blood transfusion? | 1. Yes 2. No |  |
| 120 | How much unit did she transfused? | __________(unit) |  |
| 121 | Does the mother take an antibiotic as a prophylaxis? | 1. Yes 2. No |  |
| 122 | What is the length of incision site? | ________cm |  |
| 123 | What type of wound was it? | 1. Clean wound 2. Clean contaminated wound 3. Contaminated 4. Infected wound |  |
| 124 | What was the class of Wound? | 1. Class I 2. Class II 3. Class III 4. Class IV |  |
| 125 | How much was the pre operative hematocrit count? | -----------------% |  |
